# Fabrication of a Fully Printed Ammonia Gas Sensor Based on ZnO/rGO Using Ultraviolet–Ozone Treatment

**DOI:** 10.3390/s24051691

**Published:** 2024-03-06

**Authors:** Mijin Won, Jaeho Sim, Gyeongseok Oh, Minhun Jung, Snigdha Paramita Mantry, Dong-soo Kim

**Affiliations:** 1 Department of Creative Convergence Engineering, Hanbat National University, Yuseong-gu, Daejeon 305-719, Republic of Korea; 2 Research Institute of Printed Electronics & 3D Printing, Hanbat National University, Yuseng-gu, Daejeon 305-719, Republic of Korea

**Keywords:** fully printed, zinc oxide, reduced graphene oxide, UV–ozone treatment, ammonia gas sensor

## Abstract

In this study, a room-temperature ammonia gas sensor using a ZnO and reduced graphene oxide (rGO) composite is developed. The sensor fabrication involved the innovative application of reverse offset and electrostatic spray deposition (ESD) techniques to create a ZnO/rGO sensing platform. The structural and chemical characteristics of the resulting material were comprehensively analyzed using XRD, FT-IR, FESEM, EDS, and XPS, and rGO reduction was achieved via UV–ozone treatment. Electrical properties were assessed through I–V curves, demonstrating enhanced conductivity due to UV–ozone treatment and improved charge mobility from the formation of a ZnO–rGO heterojunction. Exposure to ammonia gas resulted in increased sensor responsiveness, with longer UV–ozone treatment durations yielding superior sensitivity. Furthermore, response and recovery times were measured, with the 10 min UV–ozone-treated sensor displaying optimal responsiveness. Performance evaluation revealed linear responsiveness to ammonia concentration with a high R^2^ value. The sensor also exhibited exceptional selectivity for ammonia compared to acetone and CO gases, making it a promising candidate for ammonia gas detection. This study shows the outstanding performance and potential applications of the ZnO/rGO-based ammonia gas sensor, promising significant contributions to the field of gas detection.

## 1. Introduction

In modern society, the use of chemical substances in industry and everyday life is increasing. Consequently, the issues caused by exposure to various chemical substances are increasing. Carbon monoxide, ammonia, nitrogen oxide, and volatile organic compounds (VOCs) are the main pollutants. Among these chemicals, ammonia is extensively used in agricultural, industrial, and environmental sectors. [1,2,3]. However, ammonia is a colorless toxic gas composed of nitrogen and hydrogen [4,5,6]. High concentrations of ammonia directly affect bodily functions and cause diseases such as diabetes, and ammonia is one of the main contributors to air pollution [7,8,9]. Particularly at 50 ppm, there is a narrowing of the throat, leading to obstruction of the upper airway. Additionally, accumulation of fluid in the lungs can occur, potentially resulting in mucosal burns in the bronchi [10]. Consequently, research aimed at developing ammonia gas detection and monitoring technologies has increased steadily [11,12]. To date, metal oxide semiconductors such as SnO_2_, ZnO, TiO_2_, and In_2_O_3_ have been primarily used as materials for detecting ammonia and other gases [13,14,15,16]. In particular, ZnO, which has a wide bandgap (approximately 3.35 eV) and excellent semiconductor properties, has been widely used in gas sensors [17,18]. However, metal oxide-based sensors generally require high operating temperatures (150–500 °C), limiting their application at room temperature [19,20,21]. Reduced graphene oxide (rGO) maintains the characteristics and chemical stability of graphene and increases conductivity via reduction. rGO enhances gas detection capabilities by strengthening the interactions with adsorbed gases owing to its large surface area [22,23]. Traditional methods for producing rGO primarily employ chemical and thermal reduction processes; however, these processes require excessive chemicals and high processing temperatures [24]. Therefore, in this study, we attempted to reduce graphene oxide (GO) using a low-temperature ultraviolet (UV)–ozone reduction process. UV–ozone treatment is environmentally friendly and enables effective reduction even at relatively low temperatures. Reduction methods that employ UV–ozone treatment have been applied in various fields such as quantum dot light-emitting diodes (QLEDs) and solar cells [25,26,27].

In this paper, we present the fabrication of a fully printed ammonia gas sensor that operates at room temperature based on ZnO and rGO via low-temperature UV–ozone treatment. The electrodes and sensing layers were printed using the reverse offset and electrostatic spray deposition (ESD) processes, respectively. The printed ZnO/GO sensors were subjected to UV–ozone treatment for 1, 3, 5, and 10 min. Consequently, increasing the UV–ozone treatment time resulted in the reduction of GO, demonstrating the formation of the ZnO/rGO composite. The reactivity of the fabricated sensor was tested by exposing it to ammonia gas at concentrations ranging from 40 ppm to 80 ppm. The highest reactivity was observed after 10 min of UV–ozone treatment, with a response of 20.70 at 80 ppm. The response and recovery times were 153 s and 79 s, respectively. The R2 value was 0.94559, which is close to 1. The sensor exhibited selective reactivity toward ammonia when exposed to ammonia, acetone, and CO gases.

## 2. Experiment

### 2.1. Preparation of ZnO/GO Composites and Characterization

Zinc oxide nanoparticle inks (Sigma Aldrich Co., St. Louis, MO, USA) and graphene oxide single-layer dispersion (Graphene Laboratories Inc., Ronkonkoma, NY, USA) were used as the sensing materials. ZnO/GO composites were prepared by adding GO of 1, 5, 10, and 20 wt% to 0.8 g of ZnO ink. Subsequently, the mixture was sonicated for 2 min using a sonicator (Kudos SK5210HP, Shanghai, China).

The characteristics of ZnO/GO were analyzed using X-ray diffraction (XRD), Fourier- transform infrared (FT-IR) spectroscopy, field-emission scanning electron microscopy (FESEM), energy-dispersive X-ray spectroscopy (EDS), and X-ray photoelectron spectroscopy (XPS). FE-SEM (HITACHI SU-8230, Tokyo, Japan) was employed to investigate the morphological and structural properties of ZnO/rGO. XRD (Bruker D8-DISCOVER, Billerica, MA, USA), FTIR (Bruker ALPHA-Drift, Billerica, MA, USA), EDS (HITACHI S-4800, Tokyo, Japan), and XPS (ThermoFisher Scientific K-Alpha+, Waltham, MA, USA) analyses were employed for investigating the crystal structure and composition.

### 2.2. Fabrication of Gas Sensor

**Figure 1:**
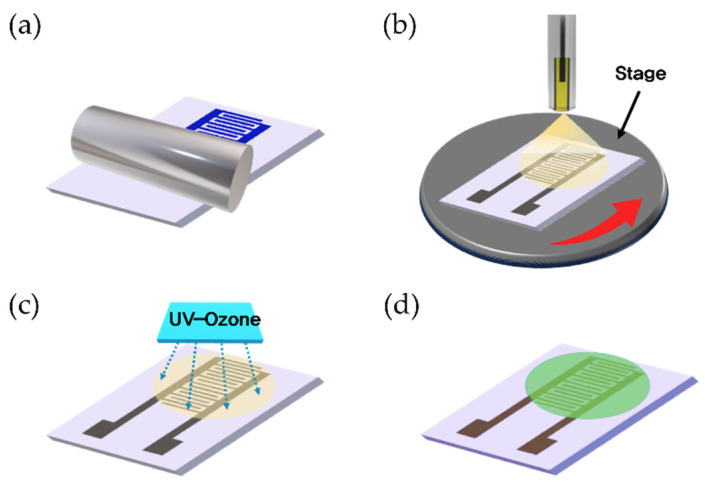
Schematic of the fabrication process of the ZnO/rGO-based ammonia gas sensor. (**a**) IDE electrode printing using reverse offset, (**b**) ZnO/GO-based sensing layer coating using ESD, (**c**) UV–O treatment for the reduction of rGO, and (**d**) fabricated gas sensor.

**Figure 2:**
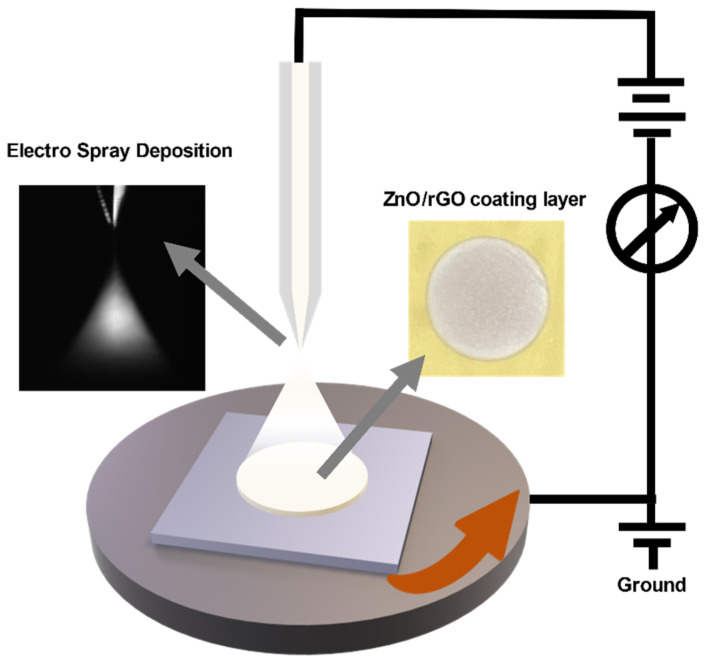
Schematic of the electrostatic spray deposition system.

Figure 1 represents an overview of the fabrication process for the ZnO/rGO-based gas sensor. A silicon wafer was used as the substrate. The electrodes were printed using Ag nanoparticle ink (DGH 55-HTG, ANP Co., Ltd., Sejong, Korea) in an interdigitated electrode (IDE) structure via a double-layer blanket (DLB) reverse offset process. The dimensions and channel length of the IDE were 7 × 14 mm and 122 μm, respectively. For electrode printing, spin coating was performed at 5000 rpm for 20 s, and the off-and-set process conditions were the same as those of previous studies [28]. The printed electrodes were then dried at 150 °C for 25 min and annealed at 400 °C for 25 min. The ZnO/GO composite was printed using the ESD process, as shown in Figure 2. In the ESD technique, a solution is injected into an electrostatic spray nozzle that sprays fine droplets onto the substrate using the electrostatic force generated by the applied voltage [29]. When voltage is applied to the nozzle, an extremely intense electric field is generated at the tip owing to the electric field focusing effects. The generated electric field penetrates the interior of the nozzle containing the solution, creating an electric double layer on the surface of the solution at the tip of the nozzle. Ions possessing double charges accumulate on the surface, resulting in surface instability, which in turn leads to the spraying of fine droplets. The nozzle diameter and distance between the nozzle and substrate were 58 μm and 16 mm, respectively, and the stage speed was 0.1 rpm. ZnO/GO was sprayed for 12 s at a voltage of 4.40 kV and then dried at 150 °C for 5 min to form a thin film. The ZnO/GO thin film was treated with UV–ozone using a UV–ozone generator (AHTECH AC-6, Anyang, Korea) for the reduction of rGO. The UV–ozone treatment times were 1, 3, 5, and 10 min, and the treatment was performed at room temperature. The final ZnO/rGO-based ammonia gas sensor was fabricated by following the aforementioned steps.

### 2.3. Measurements

The electrical characteristics of ZnO/rGO were analyzed using a source measure unit (Tektronix Keithley 2400, Beaverton, OR, USA) using the I–V curve. The ammonia gas detection performance of the sensor was measured using a mass flow controller (Phocus&SenFlow MFC, Seoul, Korea). Measurements were performed at 25 °C by placing the sensor on a platinum electrode connected to a Teflon chamber. The resistance changes in the sensor were recorded using the source measure unit. The concentration of ammonia gas was used in a range of approximately 20 to 80 ppm based on the carrier gas. Ammonia gas was injected into the chamber after ventilating it with the carrier gas. The reactivity and sensitivity of the sensor are calculated using Equations (1) and (2), respectively. R_air_ denotes the resistance when exposed to dry air, and R_gas_ denotes the resistance when exposed to ammonia gas. The response/recovery time of the sensor is defined as 90% of the total resistance change.

Response = R_gas_/R_air_ (1)

Sensitivity = (R_gas_ − R_air_)/R_gas_ × 100 (2)

## 3. Results

### 3.1. Structural and Morphological Characteristics

**Figure 3:**
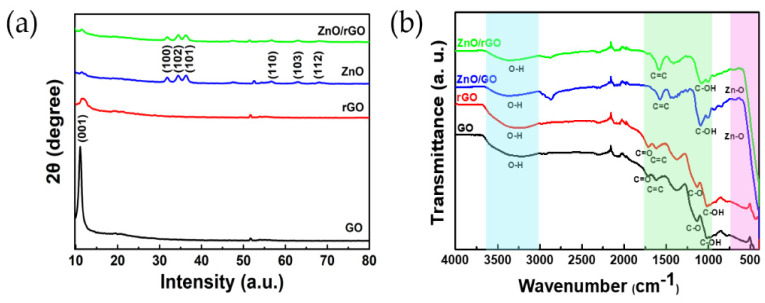
(**a**) XRD patterns of GO, rGO, ZnO, and ZnO/rGO; (**b**) FT-IR spectra of GO, rGO, ZnO/GO, and ZnO/rGO.

Figure 3a displays the XRD patterns of GO, rGO, ZnO, and ZnO/rGO, respectively. GO exhibits a high-intensity peak at 2θ = 10.96°, which can be attributed to the (001) plane [30,31]. Upon reduction from GO to rGO, the peak intensity decreases with a slight shift. The XRD pattern of ZnO illustrates peaks at (100), (102), (101), (110), (103), and (112), with corresponding diffraction angles of 31.78, 34.4, 36.34, 56.5, 62.9, and 68.14, respectively. All observed peaks for ZnO match the hexagonal wurtzite structure of ZnO (JCPDS card, No. 36-14510). The XRD pattern of the ZnO/rGO composite confirms the formation of a hybridized structure.

Figure 3b shows the FT-IR spectra of ZnO, ZnO/GO, and ZnO/rGO. A broad peak around 3000–3700 cm^−1^ is observed in all the samples. These peaks are related to the stretching vibration of a hydroxyl group (O–H bond) [32]. The FTIR spectrum of GO shows the characteristic peaks around 1724, 1622, and 1140 cm^−1^, corresponding to the vibrations of the carboxylic (C=O), aromatic (C=C), and C-O stretching vibration of an epoxy group [33]. A peak of the C–OH stretching vibration of GO is observed at approximately 1025 cm^−1^ [34]. The peaks at 1712, 1618, 1128, and 1021 in rGO are attributed to C=O, C=C, C-O, and C-OH stretching vibrations. The stretching vibrations observed at 595 cm^−1^ and 590 cm^−1^, corresponding to a Zn–O bond, confirm the presence of ZnO in ZnO/GO and ZnO/rGO. For ZnO/GO, the peaks arising from C=C and C-OH stretching vibrations are located at approximately 1588 cm^−1^ and 1094 cm^−1^, respectively, while they occur at 1579 cm^−1^ and 1080 cm^−1^ for ZnO/rGO [35,36]. These observations imply that the reduction of GO occurred due to the UV–ozone treatment. The presence of absorption peaks related to hydroxyl, carboxyl, and epoxy groups confirms the formation of the GO/ZnO and ZnO/rGO nanocomposites.

**Figure 4:**
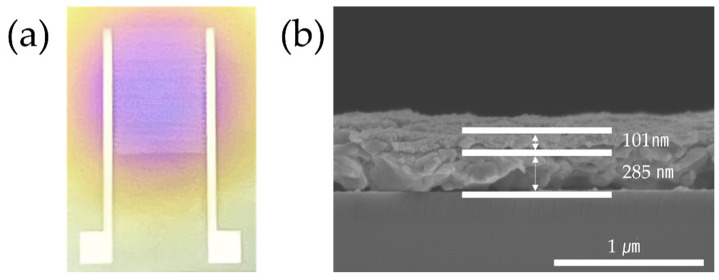
(**a**) Photographs of the ammonia gas sensor. (**b**) SEM image of the sensor (side view).

Figure 4a shows the printed ammonia gas sensor. The circular area contains a ZnO/rGO-based detection layer coated with ESD. In Figure 4b, the electrode thickness is identified as 285 nm, and the thickness of the ZnO/rGO detection layer is displayed as 101 nm.

**Figure 5:**
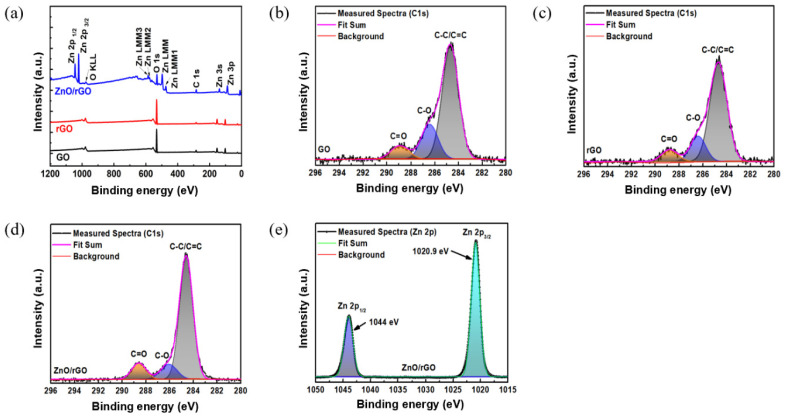
(**a**) XPS survey scan of GO, rGO, and ZnO-rGO and C 1s spectra of (**b**) GO, (**c**) rGO, (**d**) ZnO/rGO hybrids, and Zn 2p of (**e**) ZnO/rGO.

Figure 5a shows the XPS results covering the full binding energy range of GO, rGO, and ZnO/rGO. Figure 5b–d show the C 1s spectra featuring binding energy peaks in the range of 296–280 eV. At 288.9 eV, 286.4 eV, and 284.6 eV, GO exhibits the presence of C=O, C-O, and C=C/C-C (attributed to π-bonded carbon atoms), respectively [37]. Conversely, at relatively lower energies (288.7 eV, 286.3 eV, and 284.6 eV), rGO exhibited C=O, C-O, and C=C bonds, respectively [27]. This suggests that GO was stably reduced to rGO via the UV–ozone treatment. ZnO/rGO exhibits relatively lower binding energies compared to rGO, with peaks appearing at 288.1 eV, 286.07 eV, and 284.6 eV for C=O, C-O, and C=C, respectively. Figure 5e shows the Zn 2p spectrum of ZnO/rGO, with two bands at 1044 and 1020.9 eV, corresponding to Zn 2p1/2 and Zn 2p3/2 peaks. Additionally, ZnO/rGO showed the presence of Zn 2s, Zn 2p, and Zn LMM, as shown in Figure 5a, confirming the successful synthesis of ZnO/rGO.

### 3.2. Electrical Properties

**Figure 6:**
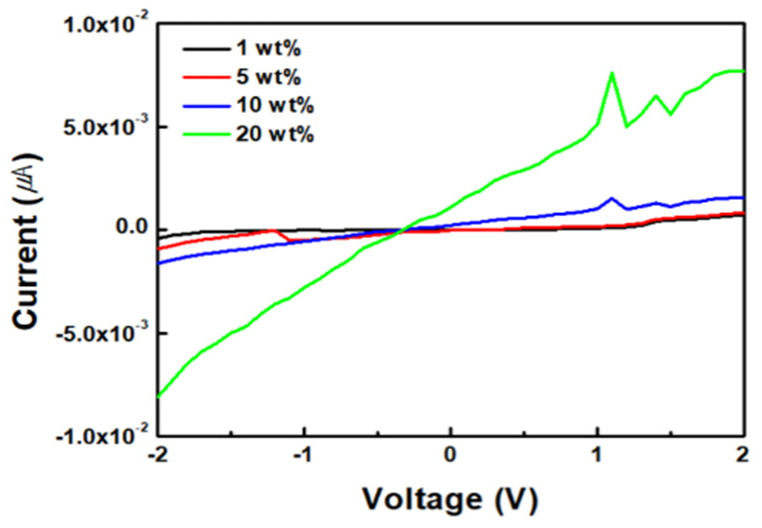
Device performance comparison of ZnO/GO at different GO concentrations.

Figure 6 illustrates the I–V curve of the ZnO/rGO sensors at approximately 25 °C. Figure 6 illustrates a graph that confirms the conductivity from −2 V to 2 V, depending on the GO content. The currents for different GO concentrations of 1 wt%, 5 wt%, 10 wt%, and 20 wt% are given as various numerical values ranging from −4.20 × 10^−4^~7.10 × 10^−4^, 9.02 × 10^−4^~8.10 × 10^−4^, −1.62 × 10^−3^~1.54 × 10^−3^, and −8.10 × 10^−3^~7.70 × 10^−3^ μA. As the GO content increased, the conductivity increased proportionally, demonstrating an enhanced conductivity at 20 wt% GO. 

**Figure 7:**
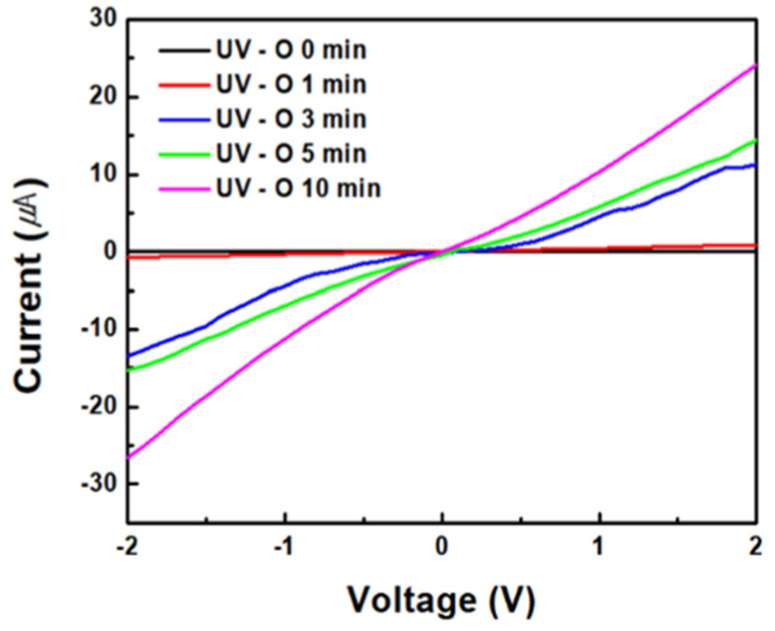
Device performance of ZnO/rGO for different UV–ozone treatment times.

Figure 7 displays the conductivity and semiconductor characteristics of ZnO/rGO at 20 wt% GO from −2 V to 2 V, based on the duration of UV–ozone treatment. The currents for ZnO/rGO at different UV–ozone treatment durations of 0 min, 1 min, 3 min, 5 min, and 10 min range between −1.62 × 10^−2^~1.54 × 10^−2^, −7.92 × 10^−1^~7.70 × 10^−1^, −1.34 × 10^−1^~1.13 × 10^−1^, −1.53 × 10^−1^~1.44 × 10^−1^, and −2.66 × 10^−1^~2.41 × 10^−1^ μA. The samples exhibit ohmic characteristics typical of p-type semiconductors. As the UV–ozone treatment time increased, the oxygen from GO was removed, thereby improving the electrical properties of rGO. Additionally, the treatment enhanced the p-type characteristics of rGO, suggesting that the n-type characteristics of ZnO became more pronounced through the formation of a heterojunction by enhancing the charge mobility.

### 3.3. Gas Sensing Properties

**Figure 8:**
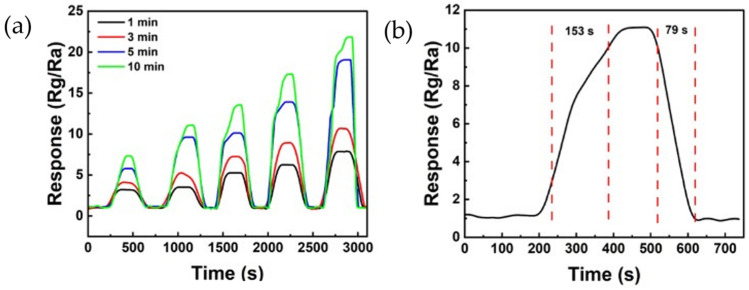
(**a**) Response curve of the ZnO/rGO-based sensor to various ammonia concentrations of 40 ppm, 50 ppm, 60 ppm, 70 ppm, and 80 ppm, respectively, at room temperature. (**b**) Response and recovery curves of ZnO/rGO using UV–ozone for 10 min to 50 ppm ammonia at room temperature (25 °C).

Figure 8 shows the reactivity of the ZnO/rGO ammonia gas sensor. Figure 8a illustrates the response curve measured by exposing the sensor to ammonia concentrations ranging from 40 to 80 ppm at 25 °C. The sensor treated with UV–ozone for 1 min showed responses of 3.19, 3.50, 5.26, 6.23, and 7.84 at 40, 50, 60, 70, and 80 ppm, respectively. The sensor treated with UV–ozone for 3 min displayed responses of 4.06, 5.17, 7.23, 8.90, and 10.66 at 40, 50, 60, 70, and 80 ppm, respectively. The sensor treated with UV–ozone for 5 min exhibited responses of 5.56, 9.41, 9.91, 13.69, and 18.83 at 40, 50, 60, 70, and 80 ppm, respectively. Finally, the sensor treated with UV–ozone for 10 min showed responses of 7.23, 10.96, 13.42, 17.18, and 21.70 at 40, 50, 60, 70, and 80 ppm, respectively. The sensor treated with UV–ozone for 10 min exhibited the highest reactivity. Additionally, as the duration of the UV–ozone treatment increased, the response also increased, clearly indicating a change in relation to ppm. Figure 8b shows the response time and recovery time measured after exposing the sensor treated with UV–ozone for 10 min to 50 ppm of ammonia at 25 °C. The response time was 153 s, and the recovery time was 79 s.

Table 1 provides a comparative analysis of various ammonia gas sensors. Metal oxide semiconductors are characterized by their ability to operate at high temperatures. For example, ZnO, among various metal oxide semiconductors, exhibits an operating temperature of 400 degrees, necessitating the incorporation of additional heaters in practical applications [18]. To address the challenges posed by elevated operating temperatures, the utilization of polymer materials such as MWCNT and rGO enables detection at room temperature without the requirement for supplementary heating. However, these composite materials manifest low response values, specifically 1.022 and 3.05. Moreover, their fabrication entails multistep deposition processes and dropcast, as opposed to a streamlined printing process, resulting in substantial material consumption and a complex production procedure [38,39]. Even in the case of exclusive rGO utilization, the outcomes reveal detection capabilities exceeding 200 ppm [40]. For achieving low ppm detection, the incorporation of metal oxides like ZIF and WO3 facilitates detection even at concentrations as low as 40–50 ppm. Nonetheless, in such instances, a fully printed process was not employed [41,42]. This paper introduces the development of a fully printed ammonia gas sensor utilizing reverse offset and ESD, showcasing exceptional reactivity. Furthermore, in the application of rGO, distinct from other studies employing chemical or thermal reduction methods, UV-ozone was employed for reduction, underscoring efficiency and innovation in comparison to existing research on rGO-based sensors.

**Figure 9:**
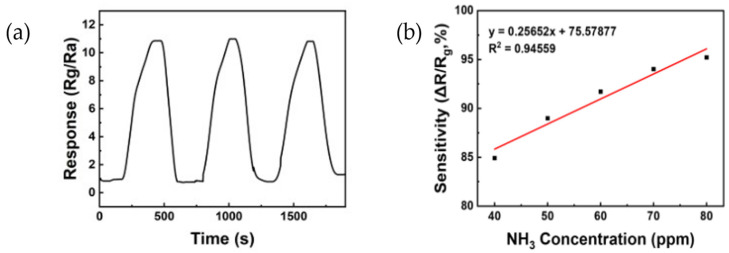
(**a**) The reproducibility of ZnO/rGO using UV–ozone for 10 min to 50 ppm ammonia at room temperature (25 °C). (**b**) Linear response to various ammonia concentrations in the range of 40 to 80 ppm.

Figure 9 shows the reproducibility and linearity of the ZnO/rGO gas sensor treated for 10 min with UV–ozone. Figure 9a displays the reproducibility measured in a 50 ppm ammonia gas treated with UV-ozone for 10 min. Through three repetitions, it is evident that the sensor consistently responds with values similar to the initial response, indicating a stable and reliable reaction. Figure 9b shows the linearity between sensitivity and ammonia concentrations ranging from 40 to 80 ppm. The linearity was evaluated through a regression analysis described by Equation (3), where y represents the resistance and x represents the injected gas concentration. The value of R^2^ (indicative of the model fit) was 0.94559. This value is nearly equivalent to 1, which suggests that the sensor possesses both reactivity and stability.

y = 0.25652x + 75.57877 (3)

**Figure 10:**
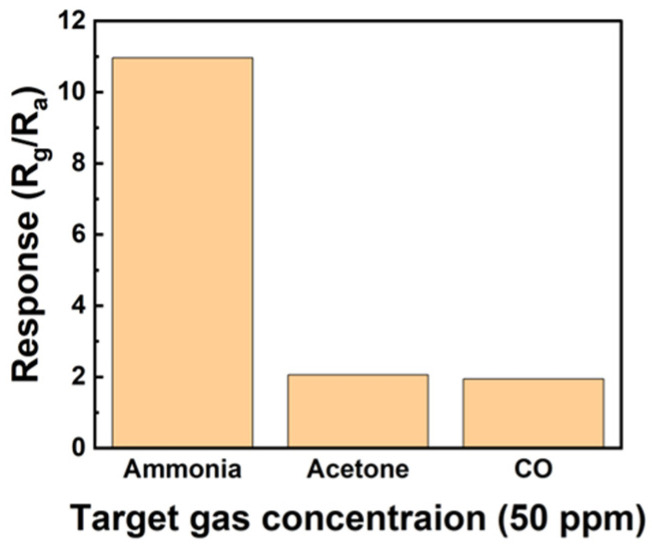
Selectivity of the sensors based on ZnO/rGO toward 50 ppm gases, including ammonia, acetone, and CO.

Figure 10 shows the selectivity through a response graph obtained after exposing the sensor to 50 ppm ammonia, acetone, and CO gas. The sensor exhibited responses of 10.96, 2.06, and 1.94 to ammonia, acetone, and CO gas, respectively. The sensor exhibited the highest reactivity to ammonia, suggesting that it is well suited for use as an ammonia gas sensor.

## 4. Discussion

A gas sensor based on the p–n heterojunction operates by employing one of the materials as a p-type or n-type semiconductor, thereby forming a junction at the interface. The interfacial area and charge transfer at this interface play a pivotal role in the performance of the gas sensor [43]. The mechanism of the ZnO/rGO gas sensor employed in this experiment for detecting ammonia is as follows.

**Figure 11:**
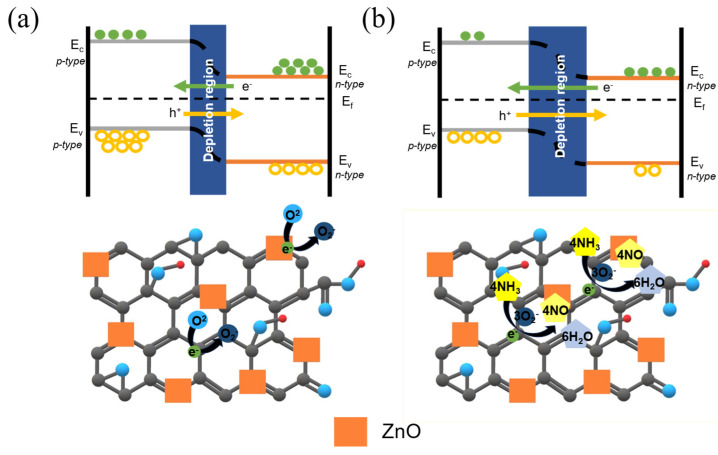
Energy band diagrams and sensing mechanisms in (**a**) dry air and (**b**) ammonia gas.

Figure 11a depicts the outcomes when the ZnO/rGO composite is exposed to dry air. Oxygen within the dry air adheres to the composite’s surface, generating oxygen species while carrying electrons, as described in Equation (4). These oxygen species establish a space charge layer at the interface and junction, offering a conduit for charge transfer and consequently reducing electrical resistance. Moreover, an electrical charge flow occurs between the p-type and n-type semiconductor layers, causing electrons to migrate from the n-type to the p-type and holes to move in the opposite direction. The alteration in electron concentration at the junction results in the formation of a potential energy barrier at the p–n junction interface [42,43].

Figure 11b illustrates the outcomes when the ZnO/rGO composite is exposed to ammonia gas. Oxygen species attached to the surface react with ammonia gas, releasing electrons as free carriers and contributing to a lower conduction band of ZnO, as outlined in Equation (5). The free electrons interact with the holes in rGO, leading to electron–hole recombination. As positive charge carriers (holes) are generated to maintain charge equilibrium due to the neutralization of the rGO material, the positive charge density increases. Within the ZnO/rGO depletion region, the positive charge density rises, and because the depletion region restricts charge movement, the relative electron density diminishes owing to increased holes. Recombination of relatively abundant holes and fewer electrons occurs in the depletion region, causing a decrease in electron density and resulting in the wider formation of the depletion region. This consequently leads to an elevation in sensor resistance.

O^2^ + e^−^ → O^2−^ (4)

4NH_3_ + 3O_2_^−^ → 6H_2_O + 2N_2_ + 6e^−^ (5)

## 5. Conclusions

We developed an ammonia gas sensor based on ZnO/rGO that is fabricated via UV–ozone treatment. Electrodes and sensing layers are printed by employing reverse offset and ESD processes. Moreover, we have analyzed the sensor characteristics as a function of the UV–ozone treatment time. As the UV–ozone treatment time is increased, the reduction of GO is improved, and the ZnO/rGO sensor exhibits a high detection ability for ammonia gas. The ZnO/rGO sensor treated with UV–ozone for 10 min exhibited a response of 20.70 to 80 ppm of ammonia gas. Furthermore, the response time and recovery time after exposure to 50 ppm of ammonia gas are 153 s and 79 s, respectively. The R^2^ value of the sensor is 0.94559, indicating a linear relationship with gas concentration. Lastly, the sensor demonstrates high reactivity to ammonia in selectivity tests against acetone and CO gas. These results validate the performance and stability of the ZnO/rGO-based ammonia gas sensor developed via UV–ozone, suggesting its potential application for practical gas detection.

## Figures and Tables

**Table 1:** Comparison with other ammonia gas sensors.

Materials	Type	Concentration (ppm)	Operating Level (°C)	Response	Fabrication Method	Reduction Method	References
ZnO	Metal oxide	250	400	1.82	Sputtering and nebulizer spray pyrolysis		[18]
rGO	Conducting polymer	200	RT	5.5	Deposition and dip coating	Chemical reduction	[38]
ZnO-MWCNT	Heterojunction	10	RT	1.022	Sputtering and chemical pyrolysis spray		[39]
ZIF/rGO	Heterojunction	50	RT	4.77	DropSens and dropcast	Chemical reduction	[40]
rGO/WO_3_	Heterojunction	40	35	8.03	Spin coating	Chemical reduction	[41]
ZnO/rGO	Heterojunction	50	RT	3.05	Thermal evaporation and spray coating	Thermal reduction	[42]
ZnO/rGO	Heterojunction	50	RT	10.96	Fully printing (reverse offset and electrostatic spray deposition)	UV-ozone reduction	This work

## Data Availability

Data are contained within the article.
